# Depression and Obesity—Do We Know Everything about It? A Narrative Review

**DOI:** 10.3390/nu16193383

**Published:** 2024-10-04

**Authors:** Jan Dębski, Józef Przybyłowski, Klaudia Skibiak, Maria Czerwińska, Maciej Walędziak, Anna Różańska-Walędziak

**Affiliations:** 1 Medical Faculty, Collegium Medicum, Cardinal Stefan Wyszyński University in Warsaw, 01-938 Warsaw, Poland; nuo.233@gmail.com (J.D.); j.przybylowski@student.uksw.edu.pl (J.P.); k.skibiak@student.uksw.edu.pl (K.S.); mariaczerwinska@student.uksw.edu.pl (M.C.); 2 Department of General, Oncological, Metabolic and Thoracic Surgery, Military Institute of Medicine—National Research Institute, Szaserów 128 St., 04-141 Warsaw, Poland; 3 Department of Human Physiology and Pathophysiology, Faculty of Medicine, Collegium Medicum, Cardinal Stefan Wyszyński University in Warsaw, 01-938 Warsaw, Poland; aniaroza@tlen.pl

**Keywords:** obesity, depressive disorders, behavioral therapy, mood disorders

## Abstract

Introduction: Due to similarities in their pathophysiology and common psychological background, depressive disorders and obesity often occur simultaneously. The treatment of obesity can reduce the symptoms of comorbid depression and, conversely, treating depression can improve weight reduction outcomes. Purpose of this study: This review aimed to analyze the available literature on the subject of various methods of treating obesity and comorbid depression and to demonstrate the mutual correlation between the therapy of depressive disorders and the therapy of obesity. Method: The Pubmed and Cochrane databases were searched for original articles on the subject of simultaneous depression and obesity that had been published between 2014 and 2024, using the key words “depression”, “depressive symptoms”, “obesity”, and “behavioral therapy”. Results and conclusions: The successful treatment of depression can help in treating obesity, especially in motivating patients to adjust their lifestyle by changing dietary habits and increasing their physical activity, which contribute to both changes in body mass index scores and reductions in depressive symptoms. Changes in self-perception, reduced daily stress, and dietary changes, as well as increased physical activity, contribute to both weight loss and the reduction of depressive symptoms. Depression and obesity should be treated as one two-dimensional disorder to achieve better long-term treatment results.

## 1. Introduction

Depression and obesity are one of the most common lifestyle diseases, the incidence of which can be called a pandemic [1,2,3,4]. It is predicted that, by 2030, as many as 1 billion people will struggle with obesity, and depression already affects over 300 million people worldwide [3]. These diseases occur in all age groups [4,5,6,7]. Women are much more likely to suffer from both obesity and depressive disorders [8], with pregnancy being a risk factor for developing both [9]. Obesity is a disease characterized by the excessive accumulation of fat tissue, which adversely affects health and leads to numerous co-morbidities [9]. An indicator of obesity is the body mass index (BMI) ≥ 30 kg/m^2^. Patients with obesity report a deteriorated quality of life, which manifests itself both through impaired physical functions and negative changes in both mental and physical health [10]. Depressive disorders are characterized by a low mood that makes every day functioning difficult for longer than 2 weeks and may have different levels of intensity [11]. Depressive disorders are associated with negative self-perception, both in the cognitive and emotional dimensions. Low self-esteem generates emotions such as feelings of sadness or guilt, which additionally cause a feeling of fatigue and reduced concentration. Negative self-perception in patients with both a depressive symptomatology and outside factors such as a negative, socially creative picture that is linked to obesity can lead to difficulties in assessing the severity of their depressive symptoms, as it is mostly based on self-reported inventories [12]. Patients with depressive symptoms and individuals with obesity often report fatigue, regardless of the presence of chronic co-morbidities. Additionally, physical inactivity, affecting patients with either obesity or depression, is known to be correlated with fatigue and poor sleep quality [13].

Both diseases increase the risk of various chronic diseases such as cardiovascular disease and type 2 diabetes [14,15]. Obesity and depression often occur together and there is a bidirectional relationship between them. Obesity increases the risk of depression and, conversely, depressive disorders increase the risk of obesity, as they are interconnected [4]. An improper diet, a lack of physical activity, weight gain, cognitive-behavioral disorders, chronic stress, and inflammation processes in adipose tissue are some of the most important causes of the co-occurrence of these diseases [4,16,17,18,19]. Therefore, it can be assumed that the treatment of one disease would have a positive impact on the treatment of the other. There are different methods of treatment available, including cognitive-behavioral therapy [20], as well as pharmacotherapy [2,11,21,22], bariatric surgery [23,24], psychotherapy [25,26,27], and changing eating habits and physical activity [28,29,30]. The purpose of the recent multitude of different therapeutic routes is raising awareness of the existence of various forms of therapy and thus allowing for deeper consideration in choosing the optimum method that would lead to greater benefits in treatment of the two coexisting diseases. Moreover, an understanding of the wide range of mutual connections between depression and obesity will enable an in-depth understanding of the causes and determinations of the risk of their coexistence, and will also allow for the faster implementation of appropriate treatment.

### Aim of the Study

The aim of this review was to present the selected literature on the subject of the relationship between obesity and depression and to analyze the correlations between different methods of treatment of coexistent depression and obesity. The additional aim of this study was to present the close relationship between depression and obesity and the wide range of therapeutic options for these two comorbidities, in order to draw attention to the latest effective treatment programs and activities which lead to improvement in the symptoms of both obesity and depression. The analysis of the multitude of forms of treatment would possibly help patients and healthcare professionals make the right decision regarding individual therapy.

## 2. Material and Methods

PubMed and Cochrane databases were searched for original articles and systematic reviews/metaanalyses about simultaneous presence of depressive disorders and obesity that had been published between 2012 and 2024. The research was performed by three junior researchers and then supervised and verified by the senior researcher. The da-tabases were searched for key words: “depression”, “depressive symptoms”, “obesity”, and “behavioral therapy”. Articles included in the review were original retrospective and prospective cohort studies, randomized controlled trials, and systematic re-views/metanalyses, and studies based on small samples of participants or with unclear methodology were excluded from the analysis.

### 2.1. Mutual Relationship between Depression and Obesity

#### 2.1.1. Inflammation Process in Adipose Tissue

Depressive disorders and obesity may have a common origin or result from each other. Obesity is an inflammatory disease of adipose tissue that results in an altered secretion of leptin, a hormone responsible for the state of satiety after a meal. A constantly increased leptin concentration in plasma leads to tissue leptin-resistance, dysregulation of food intake, constant feeling of hunger, and, ultimately, weight gain. A recent study conducted by researchers from the Federal University of São Paulo Interdisciplinary Obesity Group Program in a group of adolescents with obesity showed that increased plasma leptin concentration was positively correlated with occurrence of depressive symptoms [31]. Moreover, the inflammation process in adipose tissue leads to increased production of pro-inflammatory cytokines, which may have a negative impact on the expression of neurotransmitters that can contribute to the development of depression [4,32].

#### 2.1.2. Chronic Stress

The most common disorder associated with depression is psychological stress. This phenomenon is well illustrated by an increase in cortisol concentration in the blood caused by the activation of the hypothalamic–pituitary–adrenal (HPA) axis. The main causes of overactivation of the HPA axis in excessive stress reactions co-occurring with depression include, among others, various traumatic experiences and changes in monoaminergic pathways. Chronic stress may be a predictor of obesity because high levels of cortisol, persisting for a long time, disturb carbohydrate metabolism, causing insulin resistance and the accumulation of visceral fat [4]. Additionally, stress increases appetite and intensifies unhealthy eating behaviors, which also contributes to the development of obesity [7].

#### 2.1.3. Sleep Disorders

Sleep disorders are also associated with obesity and depression, as they have high prevalence as co-morbidities in both diseases. Shorter sleep time increases sympathetic activity, which reduces leptin secretion, and the resulting constant feeling of hunger leads to weight gain [4]. Poor sleep is also associated with increased ghrelin levels. Ghrelin is a hormone that is physiologically released to increase appetite and food intake. Disrupted sleep may disturb carbohydrate metabolism through overactivation of the HPA axis and increased secretion of glucocorticoids. This mechanism generates stress and insulin resistance, which, respectively, contribute to the development of depression and obesity [4].

#### 2.1.4. Improper Diet and Reduced Physical Activity

A diet high in carbohydrates and reduced physical activity are common causes of both obesity and depressive disorders. An intervention study, performed in a group of adults from Seattle, was designed to measure subjective mood and energy levels on a high-glycemic load (HGL) and low-glycemic load (LGL) diet. The researchers found that high-carbohydrate meals resulted in greater intensity of depressive disorders [33]. Another study, that included overweight/obese adults of both sexes, assessed the effects of aerobic training on participants’ quality of life. The results showed significant improvement in the studied mental health disorders, measured by reduction in intensity of depressive symptoms, as well as increase in physical activity, of the participants [34]. The results from the study suggest that sedentary lifestyle and lack of physical activity might promote development of obesity and aggravate the severity of comorbid depressive symptoms.

#### 2.1.5. Neural and Eating Disorders

Emotional dysregulation and changes in cognitive control caused by neural disorders may lead to depression and eating disorders [35]. The main eating disorders include emotional eating (EE) [18], defined as excessive eating in response to negative and positive emotions, and binge-eating disorder (BED) [3,26], which involves recurrent situations of binge eating accompanied by a sense of loss of control. As a result, the patient’s body weight increases, which may ultimately lead to obesity. Additionally, neural changes like improper action of the prefrontal cortex (dLPFC) cause patients to be less interested in weight loss programs [35].

#### 2.1.6. Family Factors

Parents’ attitude towards their child’s illness has a significant impact on both obesity and depression in children and adolescents. Many studies showed that depressive disorders in parents of obese children, along with difficulty coping with problems or a history of overeating, influenced their offspring, who were not satisfactorily motivated to change their eating habits or engage in physical activity [25,26,27]. Additionally, co-occurring depressive and emotional disorders are likely to be more severe in children.

### 2.2. Methods of Treatment for Co-Existing Obesity and Depression

#### 2.2.1. Cognitive-Behavioral Therapy (CBT) and Behavioral Weight Loss Program (BWL)

Programs that use behavioral weight management techniques are mostly used to treat obesity; however, they can also be useful in improving symptoms of comorbid depression [20]. Cognitive-behavioral therapy assumes that human behavior and emotions depend on a learned pattern of reacting to the surrounding world and life situations. Even though the primary result of weight loss is usually promising, unfortunately, there is a high risk of weight regain after completing a behavioral program without long-lasting effects [20]. The Weight loss Referrals for Adults in Primary care (WRAP) study included a tool prepared to examine the impact of these programs on long-term mental health. The study was designed to evaluate behavioral weight management therapy for symptoms of depression and anxiety 5 years after baseline timepoint. Moreover, the study compared effectiveness of two weight loss programs, 12 weeks long and 52 weeks long, after 5 years in relation to the baseline value. After 60 months, most participants in both the 52-week and 12-week programs had a significant reduction in the prevalence of depression symptoms [20]. Additionally, participants with more intense depressive disorders before their introduction to the program had better results from the intervention [36,37]. A randomized controlled trial compared the impact of behavioral therapy, implemented before a lifestyle change, on the depressive symptoms and efficacy of weight loss after 6 months and 12 months between a group of participants which underwent only lifestyle intervention (LI) and a group which underwent LI complemented with behavioral therapy (LI-BA). The results showed that the LI-BA group had a greater reduction in the prevalence and intensity of depressive symptoms than those in the LI group, with no significant differences in weight loss between the groups [38].

#### 2.2.2. Digital Solutions

Digital solutions are also increasingly used to improve physical, mental, and behavioral health [39]. According to a recent study, online cognitive-behavioral therapy (iCBT) also proved to be effective in improving mental health. It was used as a component of a study aimed at examining the correlation between BMI changes and the results of previous treatment with this method. Despite finding that the BMI reduction was not significant, primarily obese participants had a greater reduction in depressive symptoms than those who were not obese [40]. Additionally, smartphone applications were also created as a digital form of behavioral therapy (CBT) [39,41]. Using these tools, patients were able to monitor their weight, the food they ate, and their engagement in physical exercises. Additionally, participants remained in constant contact with the trainers and had access to various health-related materials. A randomized trial showed that technological behavioral therapy solutions resulted in significant weight loss that was maintained after 8 and 24 weeks [41]. Online behavioral therapy solutions are also used to treat obesity in stroke patients. The Swipe out Stroke study was conducted to compare a behavioral intervention conducted via a smartphone with one conducted in the form of a food diary. Both treatment methods were intended to influence the weight loss in obese patients after a stroke [42]. Through the application, participants could independently search for healthy meals and adjust their diet and physical activity to meet the requirements of their therapy. The results showed that both methods were effective in terms of weight loss; however, online treatment was more effective in improving depressive symptoms, which, due to the bidirectional relationship between depression and obesity, might additionally contribute to improving the results in obese patients [42].

#### 2.2.3. Influence of COVID-19 Pandemic

The COVID-19 pandemic has taken a huge toll not only on the general health but also on the mental health of the world’s population. The first months of the lockdown were particularly rich in the manifestation of various mental disorders, such as anxiety disorders and depression, which later regressed only slightly [43]. Additionally, the global incidence of depression increased by 24% in 2021 compared to 2017 [44]. The global situation also influenced the development of eating disorders, which resulted mainly from limited exercise, social isolation, inappropriate eating patterns, and fear of infection, which ultimately led to weight gain and obesity [43,44,45]. The inability to leave home additionally intensified difficulties in receiving therapy for people suffering from obesity, although it also motivated them to implement new behavioral interventions [46]. Additionally, bariatric surgery procedures were postponed for the duration of the pandemic in most countries, following the recommendations of international surgical societies.

To investigate the impact of the COVID-19 pandemic and assess the effectiveness of integrated care for adults suffering from obesity and comorbid depression, a pilot randomized trial was conducted. The intervention involved the implementation of a video-based Group Lifestyle Balance (GLB) program for weight loss and problem-solving therapy (PST), supported by antidepressants, to combat depression. It turned out that the pandemic had a huge impact on the study participants. All subjects were highly likely to be socially isolated and have less healthy behaviors. Some people’s mental health also deteriorated. Most of the subjects also showed a significant inability to control their body weight, which resulted in obesity. Furthermore, the intervention demonstrated moderate effectiveness in reducing symptoms of depression and obesity compared to standard care [44]. In order to combat obesity, which became more severe during the pandemic, an online self-help intervention was also introduced, based on the assumptions of acceptance and commitment therapy, which was intended to additionally protect the mental health of the respondents [45]. This initiative used, among others, online techniques for the self-management of eating behaviors, participation in physical activity, and psychoeducation. After 4 months, the online self-help results showed greater effectiveness in achieving weight loss than standard care. Additionally, participants undergoing the program reported better well-being, reduced symptoms of depression, and greater involvement in physical activity [45].

Obese patients infected with COVID-19 suffered from particularly severe depression. Even after bariatric surgery, they experienced major mental problems and greater levels of stress than obese individuals who had not had COVID-19 infection, and severe depressive disorders negatively affected the results of bariatric surgery, as measured by % excess weight (%EWL). In a randomized controlled study, telephone-based cognitive behavioral therapy (Tele-CBT) was used to treat psychiatric complications of COVID-19, and the impact of the method on depressive symptoms in patients one year after bariatric surgery was examined. This form of therapy turned out to be very effective, as it led to a significant improvement in the symptoms of comorbid depression and overeating during COVID-19 [47].

#### 2.2.4. Behavioral Weight Loss Therapy

In addition to depressive disorders, there are other psychological factors that may contribute to the development of obesity, one of which is defined as impulsivity—uncontrolled behavior in response to some “impulse” and food addiction (FA), defined as excessive consumption of highly palatable food, additionally maintained by addictive processes. To improve both energy management and symptoms of depression, a randomized one-year multimodal intervention, the PREDIMED-Plus trial, was carried out and included a Mediterranean diet, behavioral and mental support, and the promotion of physical activity. The results showed that, after 1 year of treatment, there was a significant decrease in BMI and food addiction, along with a reduction in impulsivity. However, participants who had more addictive behaviors had lesser reductions and required more intensive behavioral therapy [48].

Behavioral weight loss (BWL) interventions are an obesity treatment method that promotes weight loss. This treatment includes proper diet, physical activity, and developing the ability to cope with various emotional and mental problems. To investigate whether treating obesity through behavioral weight loss programs also affects depressive symptoms, a study was conducted in a group of African women with obesity, based on a 24-month program supporting a healthy lifestyle. The results showed that depressive disorders, as expressed by the Center of Epidemiological Studies Depression Scale (CES-D), were associated with a change in BMI score between the baseline timepoint and 6 months after the introduction of behavioral therapy. The result of the intervention was measured by weight reduction and improvement in depressive symptoms [49]. In an Australian study, the psychological therapy consisted of 10 h group sessions every week, and a decrease in depressive symptoms was observed after both 3 months and 12 months follow-up. Additionally, emotional eating behaviors were also reduced, and the number of hours per week spent on physical activity increased. However, no significant changes in BMI were observed [3]. Another study indicated that the addition of behavioral treatment for depression to behavioral weight loss treatment resulted in significantly improved psychological outcomes and greater weight loss [50]. Physical activity plays an important role in behavioral weight loss programs, as an increased frequency of physical activity was proven to be very effective in the treatment of obesity associated with binge eating disorders and significantly reduce depressive symptoms [51].

There is also an online form of behavioral weight loss therapy (IBWL) used in reducing symptoms of depression that are comorbid with obesity. In a recent randomized study, the efficiency of this program was compared with a social media campaign. The online treatment strategy was based on an online platform, through which participants received educational materials on reducing their caloric intake, increasing their physical activity, and altering their behavioral perspective on resolving everyday problems. The online intervention proved to be more effective than a regular social program and was associated with significant weight loss and improvement in depression symptoms [52].

#### 2.2.5. The Research Aimed at Improving Both Mood and Weight (RAINBOW) Trial and the Mindful Eating (ME)

There are many existing programs for the separate treatment of depression and obesity, with there being only a few solutions that treat both diseases at the same time [53,54]. The Research Aimed at Improving Both Mood and Weight (RAINBOW) trial [16,53,54,55] examined one of the methods of integrated therapy for obesity and depression that consisted of two programs. The Group Lifestyle Balance (GLB) program was based on treating obesity with lifestyle changes, including diet and physical activity, using film materials and cognitive assumptions as tools [53]. The second component of the therapy was the PEARLS program, which included behavioral techniques and optional pharmacotherapy for depressive disorders. This integrated approach was observed to have resulted, after 6 months, in an increased consumption of vegetables and fruits, a reduction in BMI, and improvement in depression symptoms. However, after 12 months, the effects of this method weakened, which might have resulted from binge eating disorders [14,53,54].

One of the methods used in the treatment of emotional eating disorders is “conscious eating” (ME) [18]. To assess the effectiveness of this method in the treatment of obesity and comorbid depression, a study based on a program that consisted of 8 psychotherapeutic sessions was conducted. The sessions were focused on awareness of the importance of “real” feelings of hunger, not caused by other factors, and on controlling emotional eating. Additionally, it was examined how self-criticism and compassion affect mental health. The results of the intervention showed that mindfulness in the emotional experience of food consumption influenced the selection of healthy food, and self-compassionate behavior prevented the loss of motivation and intensification of depressive symptoms. Therapy based on conscious eating primarily affected a reduction in emotional eating behaviors and contributed to the treatment of obesity and depression [18].

#### 2.2.6. Motivation, Diet and Physical Activity

Even though a healthy diet and regular physical activity significantly contribute to weight loss, patients are often not willing to make the effort to exercise and change their eating habits, especially in the presence of coexisting depression. Therefore, increasing the motivation of patients to change their lifestyle plays an important role in the treatment of both obesity and depression. Women are much more likely to suffer from coexistent obesity and depression; therefore, most solutions aimed at the treatment of both diseases are targeted at women rather than men [7,56]. Men suffering from these diseases require therapy tailored to their specific preferences. A randomized trial was conducted to examine the impact of a 3-month SHED-IT (Self-Help, Exercise and Diet using Information Technology) program, a behavioral, mental, and physical treatment for obesity and depression among men. As part of the program, participants received educational materials on the subject of weight loss and physical activity, as well as mental fitness exercises that used behavioral and psychological techniques. Additionally, the respondents had access to an internet application which educated them about the nutritional value of given food products. The initiative primarily motivated men to exercise self-control and regularity in following recommendations and setting new goals. This therapeutic approach was found to have positively motivated men to follow the diet and perform physical and mental exercises, which had a positive impact on the treatment of their obesity and comorbid depression [56].

An important aspect of treating obesity, and thus improving mental health, is ensuring an appropriate quality of the patient’s diet. Hypocaloric diets, such as those high in protein or low in fat, were proved to be a very good solution both in losing weight and in reducing depressive disorders [28]. The 6-month MEASUR-UP (Measuring Eating, Activity and Strength: Understanding the Response- Using Protein) study evaluated a weight loss intervention that increased protein intake. The dietary intervention had a significant effect in terms of both weight loss and improvement in mental health, although depression symptoms only improved after 3 months [57]. The is also a promising diet for the treatment of obesity in the elderly. A two-year diet program that included a Mediterranean diet contributed to a significant reduction in BMI and waist circumference in a group of elderly patients; however, there was no significant improvement in their depressive symptoms [9].

Physical activity is also a very important component of success in the treatment of obesity and improvement of general well-being, including the improvement of mental disorders. Recently, due to the COVID-19 pandemic, new solutions appeared in the daily practice of physical exercises for obese patients. One study consisted of exercising in virtual reality and included warm-ups and exercises such as running, swimming, and stationary cycling. The intervention was aimed at examining the impact of physical activity in virtual reality on the body mass index and depression of participants. After 8 weeks of activity, a decrease in body mass index and a significant reduction in depression symptoms were observed [29].

The most important factor determining the optimum adaptation of lifestyle changes is self-motivation, which can be strengthened through adequate therapy programs, one of which is motivational interviewing (MI). A study was conducted that assessed the results of motivational interviewing combined with nutritional psychoeducation (MINP), assuming that they would impact weight loss and improve mental health 12 months after weight loss treatment. Participants who were overweight or obese had significantly lower body mass indexes and reduced symptoms of depression because of increased motivation [30].

#### 2.2.7. Bariatric Surgery

Bariatric surgery is the mainstay of treatment for obesity as it is the only method that includes long-term results measured by %EWL and the remission of comorbidities [23]. The most popular bariatric procedures worldwide are sleeve gastrectomy (SG) and Roux-en-Y gastric bypass (RYGB). A recent randomized controlled trial with 5-year follow-up was conducted to analyze the long-term impact of bariatric surgery on mental health [24]. One year after the surgery, depressive symptoms decreased, although they were significantly increased (*p* = 0.015) by the end of the follow-up period; however, they remained on a lower level than before the surgery [24]. As the effect of bariatric surgery on weight loss is indisputable, the additional lowering of the intensity of depressive symptoms emphasizes the role of bariatric surgery in the treatment of coexistent obesity and depression.

#### 2.2.8. Deep Transcranial Magnetic Stimulation

Deep transcranial magnetic stimulation (dTMS) uses non-invasive electromagnetic stimulation of the prefrontal cortex to reduce appetite and regulate eating attitude. Additionally, dTMS modulates neurotransmitters’ levels, with a potentially beneficial effect on depressive symptoms. A study was conducted to evaluate the impact of 5-week high-frequency deep transcranial magnetic stimulation (HF dTMS) therapy on psychological changes in patients with obesity. As a result of the intervention, there was a significant decrease in BMI and impulsivity observed, with no impact on depressive disorders [1].

#### 2.2.9. Pharmacotherapy

Drug therapy can be a supportive form of treatment for both obesity and depression. Drugs used in the treatment of obesity included orlistat and naltrexone in combination with bupropion. A study was conducted in which orlistat was added to a behavioral weight loss program including patients with and without binge eating disorders (BED) to investigate its short-term effects on weight loss. Additionally, the study compared the effectiveness of both drug-free behavioral therapy and behavioral therapy combined with orlistat, with 6-month follow-up for both. There was no effect of added orlistat treatment in the BED group in terms of weight loss and, in contrast, significant weight loss was observed in the non-BED group, indicating that the treatment was more significant when behavioral therapy was combined with orlistat treatment than behavioral therapy alone. In the BED group, orlistat with behavioral therapy resulted in the significant reduction of depressive symptoms, the opposite of the results for behavioral therapy alone [21]. Another study with 6-month follow-up examined the effects of naltrexone and bupropion on weight loss in BED patients, with no significant changes in BMI or depressive symptoms being observed [22].

The introduction of analogues of glucagon-like peptide-1, for example Ozempic (Semaglutide), has become a milestone in the treatment of obesity, especially obesity associated with diabetes mellitus type 2. Semaglutide is responsible for simulating weight loss by reducing appetite [58], stimulating the satiety center in the hypothalamus, and reducing the pace of gastric emptying. A study conducted between 2019 and 2022 aimed to evaluate the effectiveness of semaglutide given once a week as an adjunct to a previously initiated lifestyle intervention in adolescents [58]. The results of the intervention showed a positive effect of the drug on weight loss and, additionally, on physical comfort [58]. Moreover, despite the overall lack of differences in mental health between the patients in the semaglutide and no-drug groups, the pharmacologically supported patients had lower rates of adverse psychiatric events than those in the placebo group [58]. Furthermore, a systematic review and meta-analysis of randomized controlled trials of semaglutide administered to adult patients with obesity and without type 2 diabetes also demonstrated a positive weight loss effect associated with subcutaneous semaglutide administration [59].

The most popular pharmacotherapy for depressive disorders is treatment with serotonin reuptake inhibitors (SSRIs), which can also be combined with other groups of antidepressants. Serotonin is a neurotransmitter that limits both food consumption and inappropriate eating behaviors such as binge eating episodes. The purpose of selective serotonin reuptake inhibitors is to increase the extracellular concentration of serotonin [60]. A study conducted to examine the relationship between baseline BMI and the effects of the pharmacological treatment of depression indicated that, for patients with BMI ≥ 35 kg/m^2^, bupropion in combination with a serotonin reuptake inhibitor had the best result in reducing depressive symptoms, as opposed to serotonin reuptake inhibitor monotherapy and venlafaxine in combination with mirtazapine [11]. However, more recent discoveries show a positive effect of fluoxetine on weight loss [60]. Nineteen randomized controlled trials, which included mainly overweight and obese women receiving various doses of fluoxetine, showed that, as the dose of this drug increased, obese patients experienced an equivalent gradual decrease in body weight. Depression scores were also lower in the group of people taking fluoxetine than in people who were not taking this drug [60].

Additionally, prebiotics and probiotics, especially Bifidobacterium and Lactobacillus strains, were observed to have had positive impacts on both obesity and depression, with a significant decrease in BMI and an increase in muscle strength. Additionally, the aforementioned bacteria activated the production of gamma-aminobutyric acid (GABA) and serotonin, which reduced the symptoms of depression [2].

#### 2.2.10. Family Therapy

One of the reasons for the observed unfavorable therapeutic results in children with obesity is a lack of parental involvement in the treatment process [25,26,27]. Parents who struggle with mental problems themselves often have difficulties in their contact with their children that lead to decreased motivation in following dietary and lifestyle recommendations [25,27]. Family intervention programs can help parents to support their children in the fight against obesity. A family intervention aimed at promoting a healthy lifestyle, Curtin University’s Activity Food and Attitudes Program (CAFAP), was aimed at teenagers and their parents to improve their attitudes towards obesity. The study was focused on setting goals and teaching parents behaviors and attitudes that would support their children in fighting obesity, with parallel physical exercise sessions to support the teenagers. The study showed that depression in adolescents was often associated with depression in their parents, and reductions in the symptoms of depression achieved throughout the program were maintained after one year of follow-up. Additionally, teenagers increased their physical activity and reduced their hypercaloric diet [27].

The More and Less study was conducted to evaluate various programs designed to help parents treat preschool-age children. Parental education influenced the development of positive attitudes towards children, which contributed to motivating children to eat healthy meals and be more physically active. Another family intervention program, KEEP (Keeping Foster and Kin Parents Supported and Trained), involved developing parents’ self-confidence and enabling parents to change their child’s behavior. This method also allowed the parents to focus more on the child by helping to solve problems, encouraging them to engage in a loving relationship with the child and responsibly control the child’s behavior. The study showed that parents’ involvement in childrens’ therapy was the best way to build good behavior and fight childhood obesity [25].

The selected articles are presented in Table 1 and Table 2.

### 2.3. Limitations of the Study

The main limitation of most of the studies analyzed was the sample size and the rate of follow-up. There were also differences between the demographic structures of study groups and study designs.

## 3. Conclusions

Based on the recent literature on the subject of coexistent obesity and depression, it can be concluded that obesity and depression are two diseases that are very closely related, with there being a high prevalence in the population [1,2,3,4,5,6,7,8]. They may have a common genesis, or one disease may become the beginning of the other one. The pathogenesis of these diseases may include both pathophysiological [4,29,30,33] and psychological mechanisms [4,23,24,25,33]. The appearance of one disease may intensify the symptoms of another coexisting disease and vice versa [4].

However, considering the multitude of therapies and treatment programs revolving around behaviorism and mental health [3,5,14,16,18,34,35,36,37,38,39,40,45,46,47,48,49,50,51,52,53], it can be concluded that there is a still-existing issue in the perceptions of both diseases. Negative self-perception and a lack of self-acceptance in patients with obesity [16] can lead to a low mood and depressive symptoms secondary to obesity. Conversely, chronic stress, feeling sadness, despair, and anxiety, and depressive symptoms [4] may contribute to emotional eating disorders [3,16,24] and thus result in the development of obesity. Cognitive behavioral therapy programs [3,5,18,34,35,36,37,38,39,40,45,46,47,48,49,50], initiatives designed to increase motivation [28,54] or conscious eating [16], are based on changing patients’ psychological attitudes and self-perception. Motivation is of the utmost importance in lifestyle-change programs, allowing consistency in regular mental and physical training along with diet [9,26,27,54,55]. Physical activity and compliance with dietary recommendations strengthen this motivation, which proves the bidirectional relationship between biological and psychological therapies. The positive effects of therapy give positive motivation for its continuation, and interventions that increase motivation may be considered as supportive treatment [28,54]. Some studies show a relationship between changes in BMI and the intensity of depressive symptoms [47]. Psychological and behavioral support should be a priority in the treatment of both depressive disorders and obesity.

## Figures and Tables

**Table 1:** How does treating obesity influence depression?

Autors	Title	Journal	Type of Article	Number of Participants
De Carvalho-Ferreira J.P. et al., 2015 [31]	Is there a role for leptin in the reduction of depression symptoms during weight loss therapy in obese adolescent girls and boys?	Peptides	Original research	75
Lasselin J. et al., 2020 [32]	Immunological and behavioral responses to in vivo lipopolysaccharide administration in young and healthy obese and normal-weight humans.	Brain Behav. Immun.	Original research	37
Molina K. et al., 2021 [7]	Psychological and behavioral pathways between perceived stress and weight change in a behavioral weight loss intervention.	J. Behav. Med.	Original research	409
Vancini R.L. et al., 2017 [34]	Pilates and aerobic training improve levels of depression, anxiety and quality of life in overweight and obese individuals.	Arq. Neuropsiquiatr.	Original research	63
Jones R.A. et al., 2022 [36]	Participant Characteristics Associated with Changes in Mental Health in a Trial of Behavioural Weight Management Programmes: Secondary Analysis of the WRAP Trial.	Obes. Facts	Original research	1267
Mallorquí-Bagué N. et al., 2021 [48]	Effects of a psychosocial intervention at one-year follow-up in a PREDIMED-plus sample with obesity and metabolic syndrome.	Sci. Rep.	Original research	342
Carson T.L. et al., 2017 [49]	Lower depression scores associated with greater weight loss among rural black women in a behavioral weight loss program.	Transl. Behav. Med.	Original research	409
Busch A.M. et al., 2013 [50]	Reliable change in depression during behavioral weight loss treatment among women with major depression	Obesity	Original research	148
Grilo C.M. et al., 2021 [51]	Physical activity changes during behavioral weight loss treatment by Latinx patients with obesity with and without binge eating disorder.	Obesity	Original research	79
Naparstek J. et al., 2017 [52]	Internet-delivered obesity treatment improves symptoms of and risk for depression.	Obesity	Original research	136
Rosas L.G. et al., 2020 [54]	Effect of an Intervention for Obesity and Depression on Patient-Centered Outcomes: An RCT.	Am. J. Prev. Med.	Original research	409

**Table 2:** How does treating depression influence obesity?

Autors	Title	Journal	Type of Article	Number of Participants
Lv N. et al., 2023 [55]	Association of Changes in Neural Targets and Dietary Outcomes among Patients with Comorbid Obesity and Depression: Post hoc Analysis of ENGAGE-2 Mechanistic Clinical Trial.	Journal of Nutrition	Original research	70
Jones R.A. et al., 2021 [37]	The impact of participant mental health on attendance and engagement in a trial of behavioural weight management programmes: secondary analysis of the WRAP randomised controlled trial.	International Journal of Behavioral Nutrition and Physical Activity	Original research	1267
Pagoto S. et al., 2013 [38]	Randomized controlled trial of behavioral treatment for comorbid obesity and depression in women: The Be Active Trial.	Int. J. Obes.	Original research	161
Sysko R. et al., 2022 [39]	An Initial Test of the Efficacy of a Digital Health Intervention for Bariatric Surgery Candidates.	Obes. Sur.	Original research	50
Heriseanu A.I. et al., 2023 [40]	The impact of obesity and overweight on response to internet-delivered cognitive behavioural therapy for adults with chronic health conditions.	Int. J. Obes.	Original research	234
Kim M. et al., 2020 [41]	Multidimensional cognitive behavioral therapy for obesity applied by psychologists using a digital platform: Open-label randomized controlled trial.	JMIR Mhealth Uhealth	Original research	70
Ifejika N.L. et al., 2020 [42]	Use of a smartphone-based mobile app for weight management in obese minority stroke survivors: Pilot randomized controlled trial with open blinded end point.	JMIR Mhealth Uhealth	Original research	36
Mueller J. et al., 2022 [45]	Supporting Weight Management during COVID-19: A Randomized Controlled Trial of a Web-Based, ACT-Based, Guided Self-Help Intervention.	Obes. Facts	Original research	388
Sockalingam S. et al., 2022 [47]	The Impact of Telephone-Based Cognitive Behavioral Therapy on Mental Health Distress and Disordered Eating Among Bariatric Surgery Patients During COVID-19: Preliminary Results from a Multisite Randomized Controlled Trial.	Obes. Surg.	Original research	81
Ma J. et al., 2019 [53]	Effect of Integrated Behavioral Weight Loss Treatment and Problem-Solving Therapy on Body Mass Index and Depressive Symptoms among Patients with Obesity and Depression: The RAINBOW Randomized Clinical Trial.	JAMA—Journal of the American Medical Association	Original research	409
Drew R.J. et al., 2021 [56]	Men’s perceptions of a gender-tailored ehealth program targeting physical and mental health: Qualitative findings from the shed-it recharge trial.	Int. J. Environ. Res. Public Health	Original research	125
Payne M.E. et al., 2018 [57]	Quality of life and mental health in older adults with obesity and frailty: associations with a weight loss intervention	Journal of Nutrition, Health and Aging	Original research	67
Serralde-Zúñiga A.E. et al., 2019 [60]	Fluoxetine for adults who are overweight or obese.	Cochrane Database of Systematic Reviews	Systematic Reviews	2216

## Data Availability

Documents containing all extracted data are available in the manuscript.
